# Enolase From *Aspergillus fumigatus* Is a Moonlighting Protein That Binds the Human Plasma Complement Proteins Factor H, FHL-1, C4BP, and Plasminogen

**DOI:** 10.3389/fimmu.2019.02573

**Published:** 2019-11-22

**Authors:** Prasad Dasari, Naile Koleci, Iordana A. Shopova, Dirk Wartenberg, Niklas Beyersdorf, Stefanie Dietrich, Alfredo Sahagún-Ruiz, Marc Thilo Figge, Christine Skerka, Axel A. Brakhage, Peter F. Zipfel

**Affiliations:** ^1^Department of Infection Biology, Leibniz Institute for Natural Product Research and Infection Biology, Hans Knöll Institute, Jena, Germany; ^2^Department of Molecular and Applied Microbiology, Leibniz Institute for Natural Product Research and Infection Biology, Hans Knöll Institute, Jena, Germany; ^3^Institute for Virology and Immunobiology, University of Würzburg, Würzburg, Germany; ^4^Research Group Applied Systems Biology, Leibniz Institute for Natural Product Research and Infection Biology, Hans Knöll Institute, Jena, Germany; ^5^Laboratorio de Inmunología Molecular, Departamento de Microbiología e Inmunología, Facultad de Medicina Veterinaria y Zootecnia, Universidad Nacional Autónoma de México, Mexico City, Mexico; ^6^Institute of Microbiology, Friedrich Schiller University, Jena, Germany

**Keywords:** complement factor H, moonlighting, immune evasion, plasminogen, blocking phagocytosis

## Abstract

The opportunistic fungal pathogen *Aspergillus fumigatus* can cause severe infections, particularly in immunocompromised individuals. Upon infection, *A. fumigatus* faces the powerful and directly acting immune defense of the human host. The mechanisms on how *A. fumigatus* evades innate immune attack and complement are still poorly understood. Here, we identify *A. fumigatus* enolase, AfEno1, which was also characterized as fungal allergen, as a surface ligand for human plasma complement regulators. AfEno1 binds factor H, factor-H-like protein 1 (FHL-1), C4b binding protein (C4BP), and plasminogen. Factor H attaches to AfEno1 via two regions, via short conserved repeats (SCRs) 6–7 and 19–20, and FHL-1 contacts AfEno1 via SCRs 6–7. Both regulators when bound to AfEno1 retain cofactor activity and assist in C3b inactivation. Similarly, the classical pathway regulator C4BP binds to AfEno1 and bound to AfEno1; C4BP assists in C4b inactivation. Plasminogen which binds to AfEno1 via lysine residues is accessible for the tissue-type plasminogen activator (tPA), and active plasmin cleaves the chromogenic substrate S2251, degrades fibrinogen, and inactivates C3 and C3b. Plasmin attached to swollen *A. fumigatus* conidia damages human A549 lung epithelial cells, reduces the cellular metabolic activity, and induces cell retraction, which results in exposure of the extracellular matrix. Thus, *A. fumigatus* AfEno1 is a moonlighting protein and virulence factor which recruits several human regulators. The attached human regulators allow the fungal pathogen to control complement at the level of C3 and to damage endothelial cell layers and tissue components.

## Introduction

The opportunistic pathogenic and saprophytic filamentous fungus *Aspergillus fumigatus* can cause invasive aspergillosis, a life-threatening disease which affects mainly immunosuppressed individuals and has a high mortality rate (1, 2). In patients with impaired lung function such as cystic fibrosis and ectopic asthma, *A. fumigatus* can also cause airway diseases like allergic asthma (AA) and allergic bronchopulmonary aspergillosis (3–5). Over the past two decades, the increase in *A. fumigatus* infections has raised the awareness for a better understanding of the interplay between *Aspergillus* and the human host. To this end, it is relevant to understand how the fungus is recognized by the intact immune system and for compromised individuals whose defective immune response results in clinical manifestations (6). Elucidating the immune escape strategies of *A. fumigatus* will allow development of new antifungal therapeutics. Inhaled conidia when reaching bronchi are immediately confronted by the host's innate immune system, including complement. Multiple soluble complement proteins and pattern recognition proteins are identified by the proteome approach in human bronchoalveolar lavage and airway fluids (7, 8).

Complement as a central part of the innate immune defense orchestrates both the humoral and cellular responses of the human host. This immediately acting cascade is activated by three pathways, the alternative pathway (AP), the classical pathway (CP), and the lectin pathway (CP) (9–11). The human host uses soluble and membrane-bound regulators to protect self-cells and surfaces from toxic complement activation products and to inhibit lysis of bystander cells (9, 10). Self-protective fluid-phase regulators factor H and factor-H-like protein 1 (FHL-1) inhibit the AP-mediated damage. Factor H is a 150-kDa plasma protein composed of 20 short conserved repeats (SCRs) (9). FHL-1, which is derived from the same gene and is encoded by an alternatively spliced transcript, shares SCRs 1–7 with factor H and has a C-terminal unique four-amino-acid extension. Both factor H and FHL-1 act as cofactors for factor I and assist in C3b inactivation, thereby accelerating the decay of the AP C3 convertase. C4b binding protein (C4BP) blocks both the CP and LP (9). C4BP, a 570-kDa plasma regulator, is composed of seven identical α-chains and one β-chain (12). The α-chain comprises eight SCRs, and the β-chain encompasses three SCRs. C4BP acts as a cofactor for factor I, which cleaves C4b and accelerates the decay of the CP/LP C3 convertases (13). C4BP acts as a cofactor for factor I-mediated inactivation of C3b in fluid phase (12, 14).

Plasminogen, a 92-kDa plasma glycoprotein, is a self-protective complement regulator. Plasminogen consists of five consecutive disulfide-bonded kringle domains which are linked to a serine protease domain (15). The N-terminal kringle domains mediate cell surface attachment, and often, lysine residues are relevant for the contact (16). Plasminogen is activated by physiological activators, i.e., tissue-type plasminogen activator (tPA) and urokinase-type plasminogen activator (uPA) to the protease plasmin (15). Active plasmin dissolves fibrin clots and degrades extracellular matrix components and membrane proteins (17). Plasmin also degrades complement proteins (15, 18, 19).

*A. fumigatus* conidia and hyphae activate all three pathways of the complement system (20, 21). Resting conidia preferentially activate the AP, whereas swollen conidia and hyphae activate the CP and the LP (22).

Similar to other fungal, bacterial, and multicellular microbial pathogens, *A. fumigatus* uses multiple strategies to control and to counteract host complement attack. *A. fumigatus* conidia and hyphae recruit the human plasma regulators factor H, FHL-1, C4BP, and plasminogen and the pattern recognition proteins pentraxin-3 and ficolin-2 (23–26). This pathogenic fungus also inactivates C3 directly. *A. fumigatus*-secreted alkaline protease 1 (Alp1) degrades the central human complement proteins C3, C4, and C5 (27). The metalloprotease Mep1 cleaves C3, C4, and C5 and inhibits all three complement pathways (28). Furthermore, the hydrophobic fungal pigment 1,8-dihydroxynaphthalene (DHN) melanin prevents C3b binding to the surface of *A. fumigatus*, thereby allowing escape from host complement attack (22). A role of complement upon *A. fumigatus* infection is concluded as C5 knockout mice show higher mortality when challenged with *A. fumigatus* (29, 30). Polymorphisms of the human (*pentraxin 3* [*PTX-3*]) *MBL (Manan binding lectin 2*) or *ficolin-2* genes increase susceptibility for *A. fumigatus* infection (25, 31, 32).

At present, 23 allergens have been identified from *A. fumigatus* (33). The allergen Aspf2, which is the ortholog of the central *Candida albicans* immune evasion protein Pra1 (pH-regulated antigen 1), is exposed on the surface of resting and swollen conidia and recruits the human plasma regulators factor H, FHL-1, and plasminogen (34, 35). The attached host regulators inhibit opsonophagocytosis and killing of conidia by human neutrophils. Aspf22, also known as enolase (AfEno1), is another *A. fumigatus* allergen, which has immune evasion potential. AfEno1-reacting immunoglobulins E (IgE) are present sera from patients with allergic bronchopulmonary aspergillosis (ABPA) (33, 36).

Enolase is a well-known microbial immune evasion and moonlighting protein. Enolase from various pathogens, i.e., the fungi *A. fumigatus* and *C. albicans*; from Gram-positive bacteria *Streptococcus pneumoniae*; from Gram-negative bacteria *Borrelia burgdorferi*; and from the parasite *Plasmodium falciparum* binds plasminogen [(37–42), Gow]. Enolase from *Leptospira interrogans* binds factor H and C4BP, whereas *S. pneumoniae* enolase binds C4BP, but apparently not factor H (3, 43). In the cytoplasm, enolase catalyzes the conversion of 2-phosphoglycerate to phosphoenolpyruvate; at the microbial surface, enolase binds human plasma proteins including plasminogen.

Given the important role of microbial enolases in immune evasion and given the role of AfEno1 (Aspf22) as allergen, we were interested to evaluate the immune evasion features of AfEno1 in more detail. Therefore, we asked if and how AfEno1 contributes to *A. fumigatus* immune evasion. To evaluate the binding potential, *A. fumigatus* AfEno1 was cloned, expressed, and purified as a recombinant protein. Recombinant AfEno1 bound plasminogen as well as factor H, FHL-1, and C4BP. AfEno1 expressed at the fungal surface contributes to immune evasion and assists in virulence. The human proteins attached to AfEno1 retain regulatory activity and inactivate complement at the levels of C3 and C3b. In addition, plasmin attached to swollen conidia damages human epithelial cells and induces cell retraction.

## Materials and Methods

### Cultivation of *A. fumigatus* and of Lung Epithelial Cells

*A. fumigatus* (ATCCY6645) strains were grown on *Aspergillus* minimal media (AMM) for 5 days at 37°C in the dark, as reported (44). Swollen conidia and hyphae were generated by incubating resting conidia in RPMI containing 10% fetal calf serum (FCS) for 4 and 8 h, respectively, at 37°C.

Human lung epithelial cells A549 (ACC 107) were cultivated in DMEM supplemented with FCS (10% v/v) at 37°C in a humidified 5% CO_2_ incubator and passaged two times per week when the cells reached confluency.

### Proteins/Antibodies

Normal human serum (NHS) was obtained from healthy donors, pooled, and stored at −80°C until use. NHS collection was approved by the ethical committee of the Medical Faculty of the Friedrich Schiller University, Jena, Germany. Purified human factor H, factor I, C4BP, C3b, C4b, and C4BP were purchased from CompTech (Complement Technology Inc., Tyler, Texas, USA). Factor H/FHL-1 deletion mutants factor H_1−5_, factor H_1−6_, factor H_1−7SFLT_, factor H_8−11_, factor H_11−15_, factor H_15−18_, and factor H_19−20_ were recombinantly expressed as described (35, 45). Plasminogen and tPA were purchased from Technoclone GmbH (Vienna, Austria). Fibrinogen and thrombin were obtained from Calbiochem. C4BP and goat human C3 antiserum and goat human factor H antiserum, goat human C4BP antiserum, and goat human C4 antiserum were purchased from CompTech (Complement Technology Inc., Tyler, Texas, USA). Goat human plasminogen antiserum was acquired from Acris antibodies (Acris Antibodies GmbH, Herford, Germany), and rabbit human fibrinogen antiserum was purchased from Calbiochem (La Jolla, USA). Horseradish peroxidase (HRP)-conjugated goat antirabbit and rabbit antigoat were from Dako (Deutschland GmbH, Hamburg, Germany). Alexa Fluor®-647-conjugated donkey antirabbit and Alexa Fluor®-488 donkey antigoat were procured from Life Technologies (Darmstadt, Germany). AfEno1 antiserum was produced by immunizing rabbits with purified recombinant AfEno1 by standard procedures (46).

### Cloning, Expression, and Purification of AfEno1

The gene of *A. fumigatus* AfEno1 with six His residues added at the N-terminus was cloned into the expression vector pET 4.1 a and expressed in *Escherichia coli* BL21 (DE3) (47). AfEno1 expression was induced with isopropyl-β-d-thiogalactopyranoside (IPTG). Bacteria were collected by centrifugation and lysed by sonication. Thereafter, the bacteria were suspended in lysis buffer containing 20 mM Tris-HCl pH = 8, 150 mM NaCl, and 10% glycerol. The lysed cells were centrifuged, and His-tagged AfEno1 was purified using Ni-NTA resin (HisTrap 5-ml columns, GE). Recombinant AfEno1 was purified from bacterial lysate, and the protein was not detected in inclusion bodies. Purity of AfEno1 was determined after separation in 10% SDS-PAGE followed by silver staining or western blotting (described below). The protein concentration was determined by Bradford assay (Bio-Rad GmbH Munchen).

### Binding Assays

Factor H, FHL-1, factor H/FHL-1 deletion mutants, C4BP, plasminogen, or gelatin [1 μg/well in 100 μl of Dulbecco's phosphate-buffered saline (DPBS)] was immobilized onto a 96-well microtiter plate at 4°C overnight. After blocking with 0.2% (w/v) gelatin in DPBS, AfEno1 at increasing amounts was added, and the mixtures were incubated for 1 h at room temperature (RT). Following washing, the wells were incubated with rabbit AfEno1 antiserum in DPBS with 3% bovine serum albumin (BSA), followed by HRP-conjugated secondary IgGs. After washing, 3,3′,5,5′-tetramethylbenzidine (TMB) (Thermo Fisher) was added, and the reaction was stopped with H_2_SO_4_. Absorbance was read at 450 nm with SpektraMax 190 (Molecular Devices).

In reverse orientation, AfEno1 or gelatin (1 μg/well in 100 μl of DPBS) was immobilized in microtiter plates. After blocking with 0.2% (w/v) gelatin in DPBS, the wells were incubated with factor H, FHL-1, C4BP, plasminogen, or with NHS for 1 h at RT. The wells were washed and bound regulators were identified as above by specific antisera against the corresponding proteins, followed by HRP-conjugated IgGs.

### Biolayer Interferometry

The binding affinity of AfEno1 and factor H, C4BP, or plasminogen were determined by biolayer interferometry (Forte Bio, Menlo Park, CA) as previously described (19, 34). For each concentration, Ni-NTA biosensors were hydrated in DPBS (0.001% gelatin) for 10 min; then recombinant AfEno1 was bound to biosensors for 120 s. After washing the sensor (30 s) with DPBS (0.001% gelatin), factor H, C4BP, or plasminogen at various concentrations were added as analytes. For each concentration, the association of the complexes was followed for 250 s. After washing the sensor, the dissociation of the complexes was followed for another 250 s. All interactions were performed at RT. As a non-binding control, heat inactivated regulators were used. The data were obtained after subtracting the buffer blank using a 1:1 model of interaction. Graphs were plotted in Graph Pad Prism 5.

### Cofactor Assay

Cofactor activity of factor H or FHL-1 bound to AfEno1 was evaluated as described (34). AfEno1 or gelatin (1 μg/well in 100 μl of DPBS) was immobilized on a microtiter plate at 4°C, overnight. After blocking the wells with 0.2% (w/v) gelatin, factor H (5, 10, and 20 μg/ml), or FHL-1 (2.5, 5, and 10 μg/ml) were added in DPBS, and the mixture was incubated for 1 h at RT. Following washing, C3b (1 μg/well) and factor I (1 μg/ml) in DPBS were added, and the mixture was incubated for 2 h at 37°C. To assess C4BP cofactor activity, C4BP (at 5, 10, and, 20 μg/ml) was added to immobilized AfEno1 or gelatin. Following washing, C4b (1 μg/well) and factor I (1 μg/ml) in DPBS were added; the mixture was incubated for 4 h 37°C. Then reaction was stopped by addition of Roti®-Load 1 and proteins were subjected to SDS-PAGE. Separated proteins were transferred to a membrane. C3b and C4b cleavage products were identified by Western blotting using goat human C3 antiserum or goat human C4 antiserum, respectively, followed by HRP-conjugated rabbit anti-goat antibodies.

### Influence of ε-Aminocaprioc Acid (ε-ACA) and Lysine Residues on AfEno1 Binding to Plasminogen

ε-ACA at increasing concentrations (0.625, 1.25, 2.5, 5, and 10 mM) and amino acids lysine, arginine, and glutamic acid at the indicated concentrations (0.375, 0.75, 1.5, 3, 6, and 12 mM) were added to plasminogen (10 μg/ml). After incubation (1 h at RT), the complexes were added to immobilized AfEno1. After further 1 h incubation at RT, bound plasminogen was identified with goat human plasminogen antiserum.

### Cleavage of the Chromogenic Substrate S2251

Plasminogen or plasminogen pre-incubated with ε-ACA (5 mM) or lysine (10 mM) was added to immobilized AfEno1. After 1 h incubation at RT and washing, the chromogenic substrate S2251 (30 μg/ml) and tPA (1 μg/ml) were added. Then the reaction was incubated at 37°C, and absorbance was measured every 30 min at 405 nm for 24 h (SpektraMax 190; Molecular Devices). In some wells also the serine protease inhibitor aprotinin (100 μg/ml) was added.

### Degradation of Fibrinogen by Plasmin Bound to AfEno1

AfEno1 (1 μg/well in 100 μl of DPBS) was immobilized onto a microtiter plate and following washing, plasminogen (1 μg/well) was added. After washing, fibrinogen (1 μg/well) and uPA (0.1 μg/well) were added, and then the mixtures were incubated at 37°C. Unbound tPA was washed off after incubation with *A. fumigatus* conidia. The reaction was stopped at the indicated time points by addition of Roti®-Load 1, and proteins were separated by SDS-PAGE and transferred to a membrane. Degraded Fibrinogen was visualized by Western blotting using rabbit antiserum for human fibrinogen, followed by goat HRP-conjugated anti-rabbit antibodies.

### Degradation of C3 and C3b by Plasmin Bound to AfEno1

Plasminogen (1 μg/well) was bound to immobilized AfEno1. Plasminogen was pure of fibrinogen. After washing, C3 (1 μg/well) or C3b (1 μg/well) and uPA (0.1 μg/well) were added. Following incubation the reaction was stopped at different time points with Roti®-Load 1, and proteins were subjected to SDS-PAGE. After transfer to a membrane, C3 and C3b degradation products were analyzed by Western blotting using goat antiserum raised against human C3.

### Flow Cytometry

To analyze AfEno1 expression on the surface of resting and swollen conidia, the conidia were incubated with rabbit anti-AfEno1 antiserum for 1 h. After washing, Alexa fluor®-488 conjugated donkey anti-rabbit antiserum was added. To determine plasminogen binding to resting and swollen conidia, plasminogen at concentrations of 2.5, 5, and 10 μg/ml was added to the conidia (2 × 10^6^) for 1 h at RT. After washing, goat antiserum raised against human plasminogen was added, followed by Alexa fluor®-647 conjugated anti-goat antiserum. After washing, fluorescence conidia were evaluated by flow cytometry (LSR II, BD Biosciences) and median fluorescence intensity (MFI) was analyzed by FlowJo.

### Indirect Immunofluorescence Assay for Plasminogen and AfEno1

Resting conidia (5 × 10^6^ cells/ml) were attached to poly-L-lysine pre-coated coverslips for 1 h at RT. To obtain swollen conidia and hyphae, the resting conidia were incubated on poly-L-lysine pre-coated coverslips for 4 and 8 h, respectively, in RPMI 1640 with 10% FCS (v/v) at 37°C. Unbound cells were washed off, and the bound cells were fixed in 4% (w/v) paraformaldehyde (PFA) in PBS for 10 min. Following blocking with 3% BSA for 1 h, plasminogen (50 μg/ml) was added to the fungal cells and the mixture was incubated for 1 h at RT. After washing, the cells rabbit AfEno1 antiserum (1:100) together with goat antiserum recognizing human plasminogen was added and incubated for 1 h at RT. Next, fluorochrome conjugated secondary antiserum was added. Following washing, the coverslips were mounted on glass slides with Mount Fluor (Pro Taqs) and images were captured with LSM 710 (Zeiss, Germany), using ZEN 2009 software.

### In-gel Fibrinogen Degradation

In-gel fibrinogen degradation was performed as described (38). Briefly, plasminogen (10 μg/ml) was added to swollen *A. fumigatus* conidia (1 × 10^6^) and then the cells were incubated for 1 h at RT. Then conidia were washed and tPA (5 μg/ml) was added. In some cases plasmin action was blocked with also the protease inhibitor approtinin (100 μg/ml). After incubation for 1 h at RT, conidia were washed, the cells were transferred to a pre-formed fibrin matrix gels. Gels were prepared by combining fibrinogen (5 mg ml^−1^), thrombin (1.25 U ml^−1^) and plasminogen (20 μg/ml) all dissolved in low melting agarose (1.25%) in DPBS. Gels were incubated in a humidified chamber at 37°C, overnight and in-gel fibrinogen degradation by plasmin bound to swollen conidia was evaluated. After incubation, the cleared agarose circles represent areas were fibrin-matrix fibrinogen was degraded. The diameter of the cleared area correlates directly with the enzymatic activity of plasmin.

### Evaluation of Cell Viability and Determination of Cell Retraction

sTo determine how swollen conidia having plasmin attached to their surface damages human lung epithelial cells, cell viability was evaluated. Human lung epithelial cells were seeded (5 × 10^4^/well) in a 96-well plate and the cells were cultivated until reaching confluence. Swollen *A. fumigatus* conidia (5 × 10^6^/well) were fixed with 4% paraformaldehyde (PFA) for 10 min. After washing, the conidia were incubated with plasminogen (200 μg/ml) for 1 h. Following washing, conidia with plasminogen bound were added to the epithelial cell monolayer and the activator tPA (50 μg/ml) was added. In some settings also the serine protease inhibitor approtinin (100 μg/ml) dissolved in FCS-free DMEM was added. The conidia were sedimented onto epithelial cells by centrifugation (200× g for 1 min). After 4 h incubation at 37°C in humidified 5% CO_2_ incubator, FCS (20 μl) was added to each well, and the cells were incubated overnight at 37°C. Then the unbound conidia were removed by washing, and resazurin (100 μl) (Promega GmbH, Mannheim, Germany) was added and cells were further incubated at 37°C for 2 h. Resorufin formation was followed at 570 nm.

Retraction of human epithelial cells from their matrix was assayed microscopically as described (19, 34). In brief, human lung epithelial cells were seeded (5 × 10^5^ cells/ well) on coverslips in a 12-well plate. Swollen conidia having plasminogen attached to their surface (25 × 10^6^/well) were treated with tPA (100 μg/ml) and added to the confluent epithelia cell monolayer in plain DMEM. The cells were co-incubated in humidified 5% CO_2_ incubator at 37°C for 2 h. Plasmin activity in some wells was blocked by aprotinin. After incubation for 2 h, unbound cells were washed off, and attached cells were fixed with PFA (4%) and stained with Texas-Red conjugated Wheat-Germ-Agglutinin (10 μg/ml; Thermo Fisher Scientific) and DAPI (10 μg/ml). The coverslips were mounted on glass slides and evaluated by confocal microscopy. Images were captured with LSM 710 (Zeiss, Germany), using ZEN software. The number of cell free spots formed and the void area was quantified by bioinformatics image analysis as described (19, 34).

## Results

### Recombinant AfEno1 Binds Factor H and FHL-1

**Figure 1:**
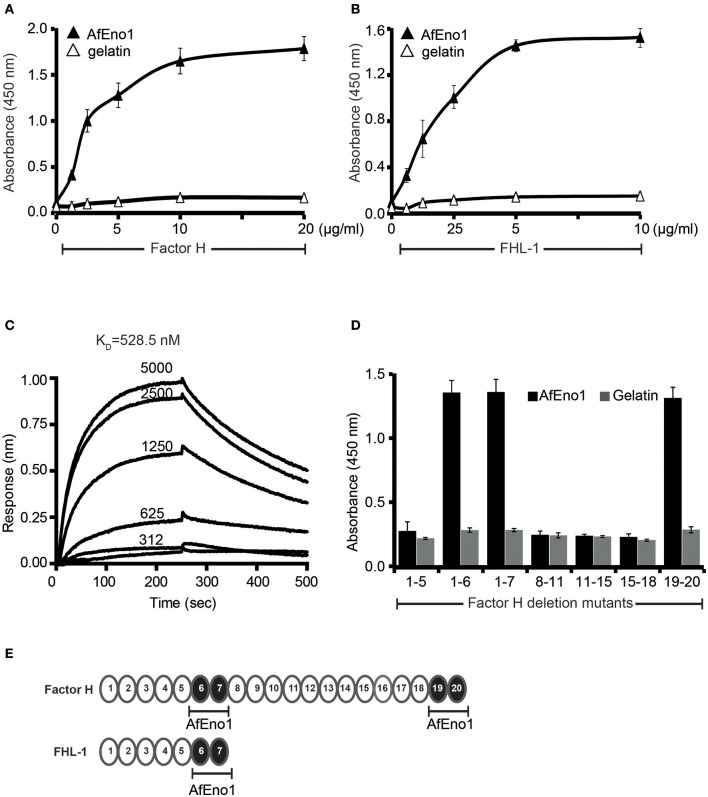
Factor H and factor-H-like protein 1 (FHL-1) bind to *Aspergillus fumigatus* enolase (AfEno1). **(A)** Factor H binds to AfEno1 dose dependently. Factor H binding to Aspf2 was assayed by enzyme-linked immunosorbent assay (ELISA). AfEno1 was immobilized onto a microtiter plate, and factor H at increasing amounts was added. After washing, bound factor H was detected with goat human factor H antiserum. Factor H did not bind to gelatin. **(B)** FHL-1 binds to AfEno1 dose dependently. FHL-1 at indicated amounts was added to immobilized FHL-1, and bound FHL-1 was detected with goat human factor H antiserum. FHL-1 showed no binding to gelatin. **(C)** Factor H binds to AfEno1 with high affinity. The affinity of factor H to AfEno1 was evaluated by biolayer interferometry. Factor H at indicated concentrations (312, 625, 1250, 2,500, and 5,000 nM) was added to AfEno1 immobilized to the Ni-NTA sensor. The association was followed for 250 s, and upon removal of the analyte, the dissociation followed another 250 s. Factor H binds to AfEno1 with a *K*_D_ = 528.5 nM. Heat-inactivated (95°C) factor H showed no binding to AfEno1 (bottom line). The experiments were repeated three times in independent assays. The results show similar association and dissociations profiles and revealed the same affinities. **(D)** Mapping of AfEno1-binding regions within factor H and FHL-1 by ELISA. Factor H/FHL-1 deletion mutants were added to immobilized AfEno1, and bound factor H/FHL-1 deletion mutants were detected by goat human factor H antiserum. AfEno1 attaches to factor H via short consensus repeats (SCRs) 6–7 and 19–20 and to FHL-1 via SCRs 6–7. **(E)** Schematic representation of SCR of factor H and FHL-1. AfEno1 binding domains of factor H (*upper*) and FHR-1 (*lower*) are highlighted in *black*. **(A,B,D)** show mean values ± SD of three separate experiments.

In order to analyze the binding repertoire of AfEno1 to human complement regulators, *A. fumigatus* AfEno1 was cloned, expressed as a His-tagged protein in *E. coli*, and purified to homogeneity (Supplementary Figures 1A–C). First, the binding of factor H and FHL-1 was studied by ELISA. When added to immobilized AfEno1, both factor H and FHL-1 bound and binding was dose-dependent (Figures 1A,B). This interaction also occurred in a reverse orientation; AfEno1 bound to immobilized factor H and to FHL-1 (Supplementary Figures 2A,B). Also, NHS-derived factor H bound to AfEno1, and again this binding was dose-dependent (Supplementary Figure 2C).

The AfEno1::factor H interaction was followed in real time by biolayer interferometry, and the affinity was determined. AfEno1 was attached to the Ni-NTA sensor, and factor H was added as analyte. The association was followed for 250 s, and upon removal of the analyte, the dissociation was followed for another 250 s. The complexes dissociated at a low rate; factor H bound to AfEno1 with a K_D_ of 528.5 nM (Figure 1C).

### Mapping of AfEno1 Binding Domains in Factor H and FHL-1

To localize the binding domains of factor H and FHL-1, factor H deletion mutants were added to immobilized AfEno1. Bound deletion mutants were detected with goat human factor H antiserum. Factor H/FHL-1 deletion fragments factor H_1−6_, factor H_1−7SFTL_ = FHL-1, factor H_19−20_ bound to AfEno1; deletion fragments factor H_1−5_, factor H_8−11_, factor H_11−15_, factor H_15−18_ did not bind (Figures 1D,E). Thus, FHL-1 attaches to AfEno1 with one region, and SCR6 seems the major contact site, and factor H binds to AfEno1 via by two regions, via SCRs6–7 with SCR6 as major contact site, which is shared with FHL-1, and via SCRs19–20.

### Factor H and FHL-1 Bound to AfEno1 Display Cofactor Activity

**Figure 2:**
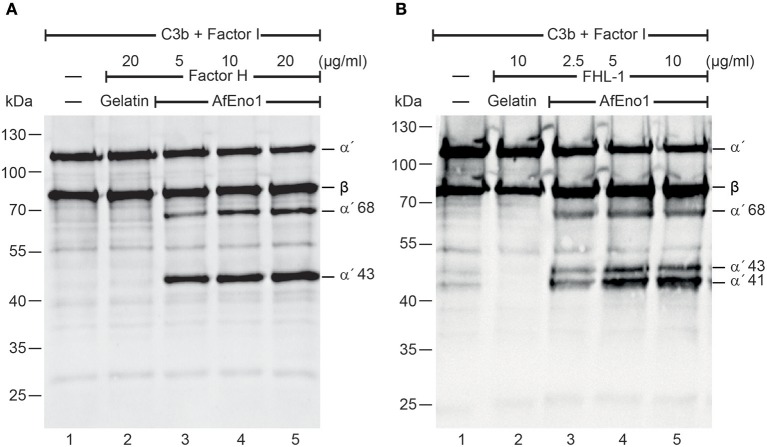
Factor H and factor-H-like protein 1 (FHL-1) bound to *Aspergillus fumigatus* enolase (AfEno1) maintain regulatory activity. **(A)** Factor H at indicated amounts was attached to immobilized AfEno1. After washing, C3b and factor I were added for 2 h and were separated by SDS-PAGE. C3b cleavage products were identified by western blotting with goat human C3 antiserum. Factor H bound to AfEno1 mediated C3b cleavage by factor I, and C3b cleavage products α′68 and α′43 kDa were identified dose dependently (lanes 3–5). When factor H was added to the negative control gelatin, no C3b cleavage products were observed (lane 2). **(B)** FHL-1 at increasing amounts was attached to AfEno1. Following washing, C3b and factor I were added for 2 h; the proteins were subjected to SDS-PAGE. After being transferred to a membrane, C3 cleavage products were visualized by western blotting. FHL-1 assisted in C3b cleavage by factor I, as revealed by the appearance of C3b cleavage products α′68, α′43, and α′41 (lanes 3–5). No C3b cleavage products were observed in the negative control gelatin (lane 2). Data shown in **(A)** and **(B)** are one representative result of three independent experiments.

We next investigated the regulatory activity of the AfEno1 attached host complement inhibitors. Factor H or FHL-1 was bound to immobilized AfEno1, and, following washing, C3b and factor I were added. Upon incubation for 2 h at 37°C, the proteins were separated by SDS-PAGE and transferred to a membrane. C3b cleavage fragments were identified by Western blotting. When bound to AfEno1, factor H assisted in factor I mediated cleavage of the α′ chain of C3b as revealed by the appearance of the α′68 and α′43 cleavage products. Cleavage was dose-dependent (Figure 2A, lanes 3–5). Similarly, FHL-1 bound to AfEno1 assisted in C3b cleavage. In this case α′68, α′43, and α′41 bands appeared. Also this effect was dose-dependent (Figure 2B, lanes 3–5). Thus, factor H and FHL-1 bound to AfEno1 retrain cofactor activity.

### Recombinant AfEno1 Binds Human C4BP, and AfEno1-Bound C4BP Retains Regulatory Activity

**Figure 3:**
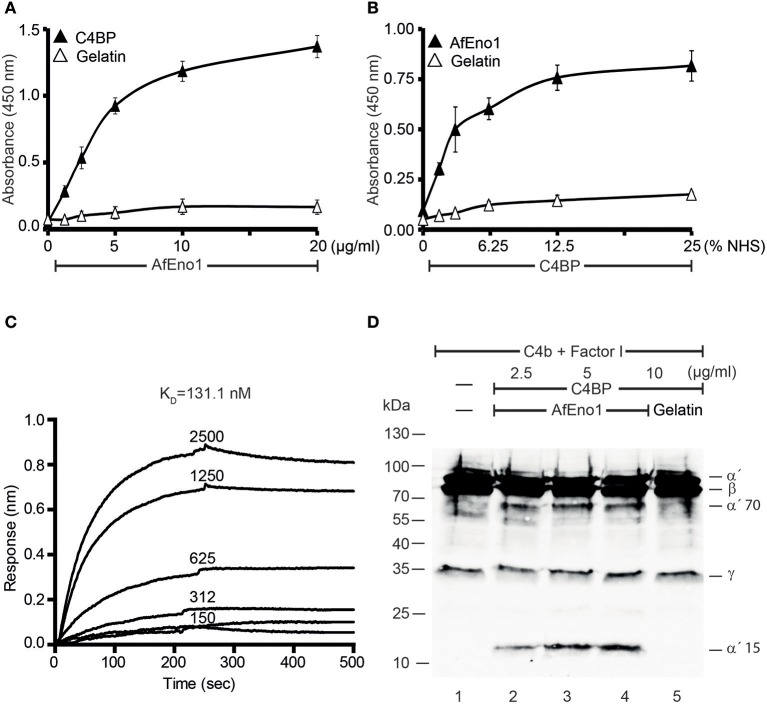
C4b binding protein (C4BP) binds to *Aspergillus fumigatus* enolase (AfEno1), and AfEno1-bound C4BP retains regulatory activity. **(A)** AfEno1 was immobilized onto a microtiter plate overnight, and C4BP at increasing amounts was added. After washing, bound C4BP was detected with goat human C4BP antiserum. C4BP showed no binding to gelatin. **(B)** Plasma-derived C4BP binds to AfEno1 dose dependently. Normal human serum (NHS) (10 mM EDTA) at different concentrations was added to immobilize AfEno1, and bound C4BP was detected as above. **(C)** The binding affinity of C4BP with AfEno1 was evaluated by biolayer interferometry. C4BP at the indicated concentrations (150, 312, 625, 1,250, and 2,500 nM) was bound to AfEno1 immobilized on Ni-NTA sensor surfaces. For each concentration, the association was followed for 250 s, and upon removal of the analyte, the dissociation followed another 250 s. C4BP binds to AfEno1 with a *K*_D_ = 131.1 nM. **(D)** C4BP bound to AfEno1 retained cofactor activity. C4BP at indicated amounts was attached to immobilized AfEno1. After washing, C4b and factor I were added for 4 h, and the proteins were separated by SDS-PAGE. After being transferred to the membrane, C4b cleavage products were visualized by western blotting with goat human C4 antiserum. C4BP bound to AfEno1 assisted in cleavage of C4b by factor I, and C4b cleavage products α′70 and α′15 were identified dose dependently (lanes 2–4). No C4b cleavage products were observed in the negative control gelatin (lane 5). **(A,B)** Are mean ± SD of three independent experiments. **(D)** Represents one of three independent experiments.

In addition, binding of AfEno1 to C4BP was analyzed by ELISA. AfEno1 bound to immobilized C4BP and binding was dose-dependent (Figure 3A). Also, NHS-derived C4BP bound to immobilized AfEno1 (Figure 3B). In reverse setting, purified C4BP also bound to immobilized AfEno1 (Supplementary Figure 2D). C4BP::AfEno1 interaction was followed in real time. To this end, C4BP was added to immobilized AfEno1, and the association was followed for 250 s. Upon removal of the analyte, the dissociation was followed for the same time. C4BP::AfEno1 complexes dissociated at a low rate. C4BP bound to AfEno1 with affinity of K_D_ 131.1 nM (Figure 3C).

We further assessed whether the regulatory activity of C4BP attached to AfEno1. C4b and factor I were added to preformed C4BP::AfEno1 complexes. After incubation at 37°C for 4 h, the proteins were separated by SDS-PAGE, transferred to a membrane and C4b cleavage was followed. C4BP bound to AfEno1 acted as cofactor for C4b degradation, and the α′70 and α′15 cleavage bands of the α′ chain appeared (Figures 3C,D, lanes 2–4).

### AfEno1 Binds to Human Plasminogen via Lysine Residues

**Figure 4:**
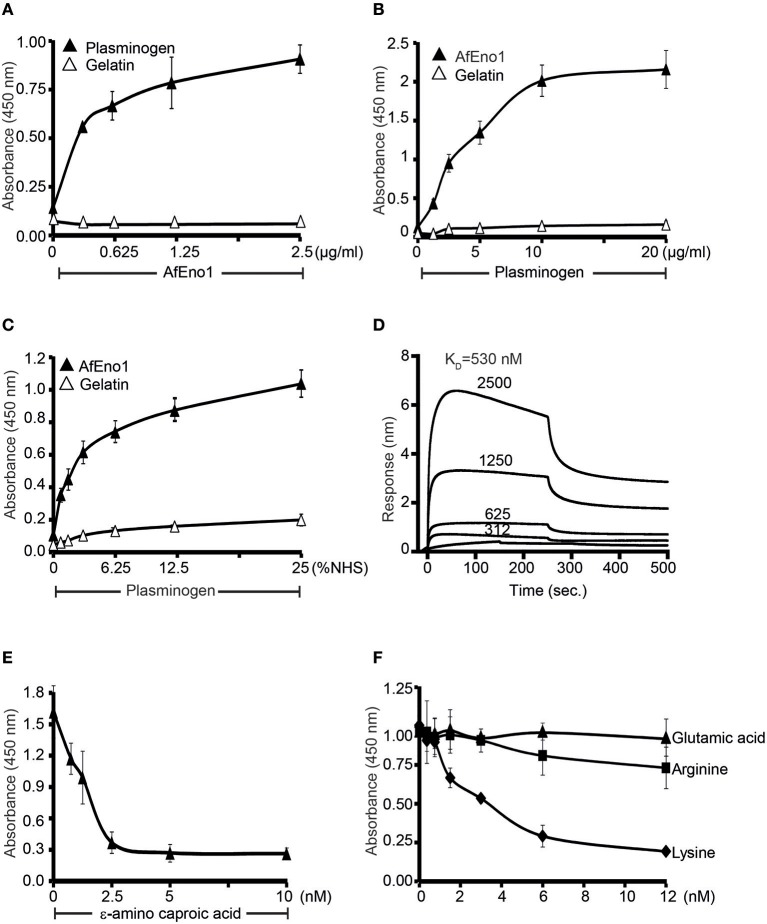
Plasminogen binds to *Aspergillus fumigatus* enolase (AfEno1) via lysine residues. **(A)** AfEno1 binding to plasminogen was assayed by enzyme-linked immunosorbent assay (ELISA). AfEno1 at indicated amounts was added to immobilized plasminogen, and bound AfEno1 was detected with rabbit AfEno1 antiserum. AfEno1 bound to plasminogen dose dependently. AfEno1 did not bind to gelatin. **(B)** Plasminogen binds to AfEno1. Plasminogen at increasing amounts was added to immobilized AfEno1, and bound plasminogen was detected with goat human plasminogen antiserum. AfEno1 showed no binding to gelatin. **(C)** AfEno1 recruits plasminogen from serum. Normal human serum (NHS) (10 mM EDTA) at different concentrations was added to immobilize AfEno1, and bound plasminogen was detected as above. **(D)** The binding affinity of plasminogen to AfEno1 was evaluated by biolayer interferometry. Plasminogen at different concentrations (312, 625, 1,250, and 2,500 nM) was added to AfEno1 immobilized on Ni-NTA biosensors. For each concentration, the association was evaluated for 250 s. Following removal of the analyte, the complex dissociation was assessed for another 250 s. Plasminogen binds to AfEno1 with a *K*_D_ = 530 nmol/L. Heat-inactivated (95°C) plasminogen showed no binding to AfEno1 (bottom line). **(E)** The lysine analog ε-ACA blocks plasminogen binding to AfEno1. ε-ACA, at the indicated concentrations, was incubated with plasminogen for 30 min, and the mixture was added to immobilized AfEno1. The bound plasminogen was detected with goat human plasminogen antiserum. **(F)** The effect of free amino acids on plasminogen binding to AfEno1 was assayed by ELISA. Lysine, arginine, or glutamic acid at increasing concentrations were added to plasminogen, and the mixture was added to immobilized AfEno1. The bound plasminogen was detected as above. Lysine blocked plasminogen bonding to AfEno1. However, arginine and glutamine acid had no effect on plasminogen binding to AfEno1.

AfEno1 binds immobilized plasminogen (Figure 4A) (42). The interaction also occurred in reverse orientation. Plasminogen bound to immobilized AfEno1, and this binding was dose-dependent (Figure 4B). Moreover, serum derived plasminogen bound to immobilized AfEno1; again in a dose-dependent manner (Figure 4C). The binding of plasminogen to AfEno1 was followed in real time. Plasminogen bound to AfEno1; binding was saturated after about 50 s, and the complex dissociated at a slow rate. Plasminogen bound to AfEno1 with a K_D_ of 530 nM (Figure 4D).

As many microbial proteins bind plasminogen via lysine residues, we asked if the lysine analog ε-aminocaproic acid (ε-ACA) influences the interaction. ε-ACA inhibited plasminogen binding to AfEno1, and the effect was dose-dependent. At 2.5 nM ε-ACA blocked binding completely (Figure 4E). In addition, lysine, but neither arginine nor glutamic acid blocked plasminogen binding to AfEno1 (Figure 4F). Thus, AfEno1 binds to plasminogen via lysine residues.

### Plasminogen Bound to AfEno1 Is Accessible for Activators

**Figure 5:**
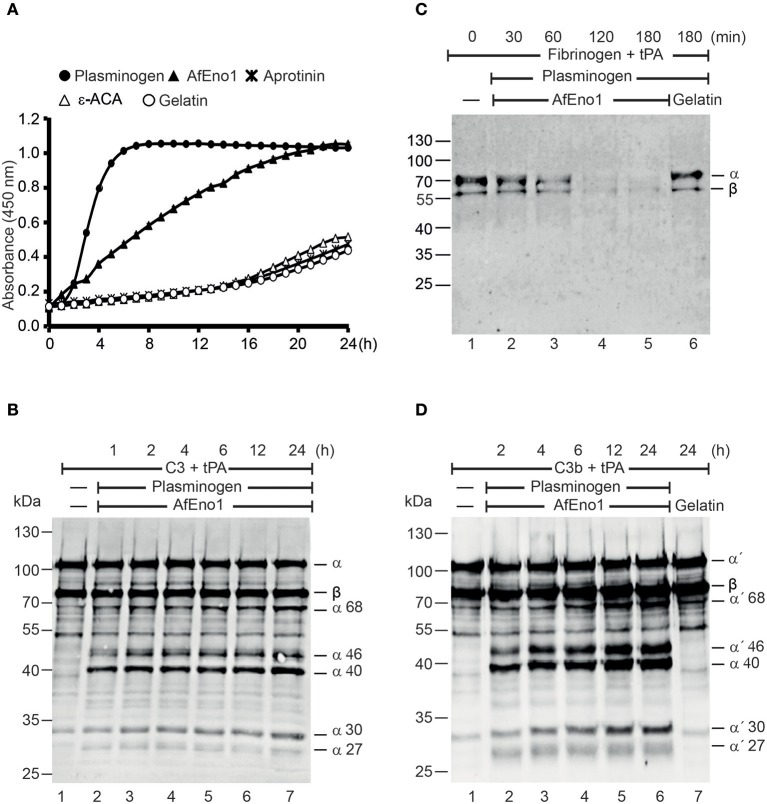
Plasminogen bound to *Aspergillus fumigatus* enolase (AfEno1) is available for the activators. **(A)** Plasminogen was added to immobilized AfEno1 on a microtiter plate for 1 h. After washing, the urokinase-type plasminogen activator (uPA) and the chromogenic substrate S-2251 were added. The plasmin activity was photometrically red at 405 nm for every 30 min, over 24 h. AfEno1-bound plasminogen was converted to plasmin, which cleaved the substrate S-2251 time dependently. ε-Aminocaproic acid (ε-ACA) and aprotinin inhibited S-2251 cleavage. No cleavage of S-2251 occurred in the negative control gelatin. **(B)** Fibrinogen and tissue-type plasminogen activator (tPA) were added to the preformed AfEno1–plasminogen complex. The reactions were stopped at indicated time points, and the proteins were subjected to SDS-PAGE. Fibrinogen degradation was visualized by Western blotting with a rabbit human fibrinogen antiserum. AfEno1-bound plasminogen was activated to plasmin which cleaved fibrinogen (lanes 2–5). No fibrinogen degradation was visualized in the negative control gelatin (lane 6). **(C)** C3 and tPA were added to the preformed AfEno1–plasminogen complex. The reaction was stopped at different time points, and the proteins were subjected to SDS-PAGE. C3 degradation products were visualized by western blotting with goat human C3 antiserum. Plasminogen bound to AfEno1 was activated to plasmin which cleaved C3 (lanes 2–7). **(D)** AfEno1 was immobilized on a microtiter plate, and after washing, C3b and tPA were added. The reaction was stopped indicated time points, and the proteins were separated by SDS-PAGE. C3b cleavage products were determined as above. AfEno1-bound plasminogen was converted to active plasmin which degraded C3b (lanes 2–7). One representative result of three independent experiments is shown in each panel.

Plasmin degrades complement proteins and extracellular matrix components (19). To assess the role of plasminogen bound to AfEno1 for immune evasion and tissue invasion, the zymogen was treated with the activator tPA. AfEno1 bound plasminogen when treated with tPA was converted to plasmin, which cleaved the chromogenic substrate S-2251 and cleavage was time-dependent (Figure 5A). No proteolytic activity was detected when the interaction was blocked by ε-ACA or in presence of aprotinin (Figure 5A). Plasmin bound to AfEno1 also degraded the physiological substrate fibrinogen. This degradation was time-dependent, and, after 2 h, fibrinogen was completely degraded (Figure 5B). Furthermore, plasmin bound to AfEno1 cleaved C3, as shown by appearance of α68, α46, α40, α30, and α27 kDa bands (Figure 5C, lanes 2–7). Also, this cleavage was time dependent. Similarly, plasmin bound to AfEno1 cleaved C3b as visualized by appearance of α′68, α′46, α′40, α′30, and α′27 kDa bands (Figure 5D, lanes 2–6). Thus, plasminogen bound to AfEno1 is accessible for tPA and activated to plasmin.

### AfEno1 Is Expressed on the Surface of Swollen Conidia and Hyphae and Colocalizes With Plasminogen

**Figure 6:**
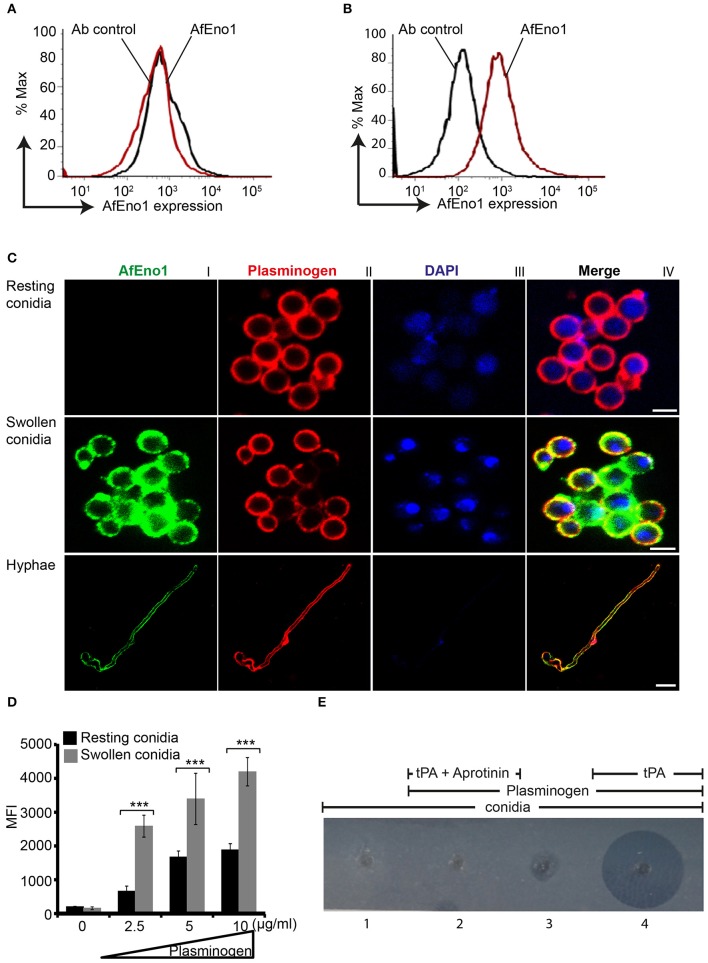
Plasminogen binds to *Aspergillus fumigatus* enolase (AfEno1) on the surface of *A. fumigatus*. Analysis of AfEno1 expression by flow cytometry. Resting conidia **(A)** and swollen conidia **(B)** were incubated with rabbit AfEno1 antiserum, followed by secondary antibodies. The cells were analyzed by flow cytometry. AfEno1 was absent on the surface of resting conidia but present on the surface of swollen conidia. **(C)** Plasminogen and AfEno1 colocalize on the surface of swollen conidia and hyphae. Purified plasminogen was added to resting conidia, swollen conidia, and hyphae. After washing, the cells were incubated with goat human plasminogen antiserum and rabbit AfEno1 antiserum, followed by corresponding secondary antibodies. AfEno1 was not expressed on the surface of resting conidia (*upper panel*). AfEno1 and plasminogen were colocalized on the surface of swollen conidia (*middle panel*) and hyphae (*lower panel*). Scale bars 2, 5, and 10 μm in upper, middle, and lower panels, respectively. **(D)** Resting conidia binds less plasminogen. Plasminogen at different amounts was incubated with resting and swollen conidia. After washing, the bound plasminogen was analyzed by flow cytometry with goat human plasminogen antiserum, followed by secondary antibodies. ***P < 0.001. **(E)** Plasmin bound to conidia degrades fibrinogen in gel. Plasminogen was bound to swollen conidia, and after washing, swollen conidia were placed in the wells of matrix gel in the presence or absence of tissue-type plasminogen activator (tPA) and the protease inhibitor aprotinin.

AfEno1 expression on the surface of resting and swollen conidia was evaluated by flow cytometry. AfEno1 was not identified on the surface of resting *A. fumigatus* conidia (Figure 6A), but was identified on the surface of swollen conidia (Figure 6B). AfEno1 was identified at high levels on the surface of swollen conidia and hyphae as shown by confocal microscopy (Figure 6C, upper panel I). AfEno1 (green fluorescence) was evenly distributed on the surface of both swollen conidia (Figure 6C, middle panel I) and hyphae (Figure 6C, lower panel I). Thus, AfEno1 is expressed on the surface of swollen conidia and hyphae, but not on resting conidia.

In addition, plasminogen (red fluorescence) distribution was evaluated. Plasminogen bound to all three morphotypes. Plasminogen colocalized with AfEno1 on the surface of swollen conidia (Figure 6C, middle panel IV) and hyphae (Figure 6C, lower panel IV). Plasminogen binding to resting conidia, which lack AfEno1 shows that *A. fumigatus* expresses additional plasminogen binding surface proteins.

### Resting Conidia Bind Less Plasminogen Than Swollen Conidia

We next compared the intensity of plasminogen binding to resting and swollen conidia. Plasminogen bound dose-dependently to both morphotypes. Plasminogen used at 2.5 μg/ml bound with high intensity to swollen conidia (MFI; 2,583 ± 323) as to resting conidia (MFI; 661 ± 150; mean ± SD, *P* < 0.0007). Thus, plasminogen bound to resting conidia with 74% lower intensity (Figure 6D).

### Plasmin Bound to Swollen Conidia Degrades Fibrinogen

To analyze the effect of plasmin attached to *A. fumigatus* conidia, we next investigated whether plasmin-attached to swollen conidia degrades a fibrinogen matrix. Swollen conidia lacked fibrinolytic activity; the fibrinogen matrix remained intact and turbid (Figure 6E, panel 1). Similarly, swollen conidia with plasminogen attached and in the absence of tPA lacked a fibrinolytic activity (Figure 6E, panel 3). However, in presence of tPA, newly generated plasmin degraded the fibrin matrix, as revealed by the clear transparent zone (Figure 6E, panel 4). Fibrinogen degradation was blocked by the serine protease inhibitor aprotinin (Figure 6E, panel 2).

### Plasmin Bound to Swollen Conidia Damages Human Lung Epithelial Cells

**Figure 7:**
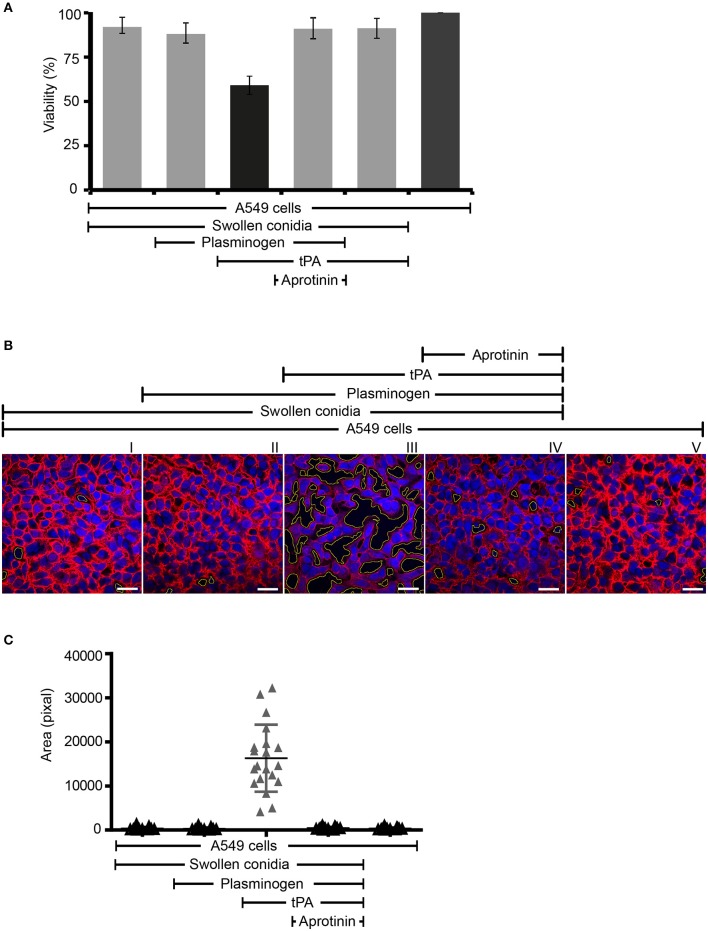
Swollen conidia-bound plasmin damages human A549 cells and induces cell retraction. **(A)** When activated to plasmin, plasminogen bound to swollen conidia damages human lung epithelial cells. Plasminogen was incubated with swollen conidia. After washing, swollen conidia were added to the monolayer of A549 cells with/without tissue-type plasminogen activator (tPA) for 24 h. After removal of the unbound swollen conidia, the cellular metabolic activity was determined by adding CellTiter-Blue®. Plasmin bound to swollen conidia decreased the metabolic activity of lung epithelial cells (column 3). This effect was inhibited by aprotinin (column 4). tPA alone has no effect on the metabolic activity of epithelial cells (column 5). Viability of human A549 cells (column 6). **(A)** is mean ± SD of three independent experiments. **(B)** Plasmin-attached swollen conidia induce cell retraction. Swollen conidia attached to plasminogen were added to the monolayer of human lung epithelial cells in the presence and absence of tPA for 2 h. After removal of the unbound conidia, cells were fixed and stained with Texas Red-conjugated wheat germ agglutinin (WGA) (red) and 4′ ,6-diamidino-2-phenylindole (DAPI) (blue). Plasmin bound to conidia induced cell retraction leading to the exposure of the extracellular matrix component. This plasmin activity was blocked by aprotinin. The area of each void spot was identified by automated image analysis and was shown in the images by green borders. Scale bars: 30 μm. **(C)** Quantification of epithelial cell damage and retraction by bioinformatics approach. The total void area of each confocal image (20 images) was quantified with novel bioinformatics image analysis. Plasmin bound to the surface of swollen conidia caused more total void area per image than the plasminogen bound to swollen conidia. Aprotinin inhibited plasmin-mediated cell damage. Horizontal bar represents mean, and vertical bar shows SD.

Next we assayed if plasmin attached to swollen conidia influences the integrity of human epithelial cells. First, plasminogen-coated conidia were added to human lung epithelial cells. Following incubation, the metabolic activity of the human cells was determined. Plasmin-attached swollen conidia damaged human A549 epithelial cells, and the metabolic activity was reduced by 41% (*P* < 0.0002) (Figure 7A, column 3). The metabolic activity of intact cells was set 100 % (Figure 7A, column 6). This effect by plasmin was blocked by the aprotinin (Figure 7A, column 4).

Next the effect on cell integrity and epithelial cell surface retraction was followed. Swollen conidia with surface attached plasminogen/plasmin were added to the epithelial monolayers. Upon incubation, cell retraction was evaluated by microscopy, and both number and area of the void spots was counted. Plasmin attached to swollen conidia induced cell retraction resulting in exposure of the underlying matrix (Figure 7B, panel III). Aprotinin blocked cell retraction (Figure 7B, panel IV). In addition, the number of spots formed and the total area of void spots was quantified by bioinformatic evaluation. Active plasmin attached to the surface swollen conidia caused cell retraction and both the number of void spots and the area of these spots was larger than spots formed by swollen conidia alone, or swollen conidia with plasminogen bound (Figure 7C).

## Discussion

Here, we identify enolase, AfEno1 also known as allergen Aspf22 as the second plasma regulator binding protein of the human pathogen *A. fumigatus*. AfEno1 binds four human plasma proteins factor H, FHL-1, C4BP, and plasminogen. AfEno1 binds factor H with a K_D_ 528.5 nM, C4BP with K_D_ 131.1 nM and plasminogen with K_D_ 530.0 nM. This affinity is comparable to that of the other *A. fumigatus* factor H ligand Aspf2 and the *C. albicans* homolog Pra1. Aspf2 binds factor H with slightly higher (K_D_ 76 nM) and plasminogen with similar affinity (K_D_ 846 nM) (34). However, *C. albicans* Pra1 binds factor H with higher affinity (K_D_ 1.87 nM) (data not shown).

Factor H and FHL-1 bound to AfEno1 assist the protease factor I in C3b inactivation. Similarly, C4BP bound to AfEno1 retains regulatory activity and assists for C4b inactivation. Plasminogen bound to AfEno1, when activated by tPA, cleaves the substrates S2251, fibrinogen, and degrades C3 and C3b.

AfEno1 binds factor H via SCRs6–7 and SCRs19-20 and FHL-1 via SCRs6-7. These domains represent common binding sites for microbial immune evasion proteins (48). These multiple microbial factor H, FHL-1 ligands, despite their common binding features, lack conserved sequence motifs (48). Factor H and FHL-1 use the same regions i.e., SCRs6–7 (factor H and FHL-1), and SCR19-20 (factor H) bind to modified self surfaces, such as necrotic and damaged human cells e.g., via glycosaminoglycans (GAGs), or modified surface constituents, like Malondialdehyde or phosphatidyl choline (49).

AfEno1 is a 438-amino-acid moonlighting protein which shares 72.6% amino acid homology with *C. albicans* enolase and which is also highly related to enolase from the microbial pathogens *Streptococcus pneumoniae, Borrelia burgdorferi, Staphylococcus aureus*, and *Paracoccidioides brasiliensis* (37–39, 41, 50). Given the sequence homology of AfEno1 with Candida enolase and with other microbial enolases and their functional homology in moonlighting it will be of interest to evaluate if all these microbial proteins, share the same immune evasion features.

Hijacking human plasminogen by fungal and microbial pathogens is a common mechanism as active plasmin promotes cell adhesion, degrades cell layers, and matrices allowing dissemination during infection (39, 51). Acquisition of plasminogen by surface expressed enolase has been show for several pathogens, including the fungal pathogens, *A. fumigatus, C. albicans*, and for the microbial pathogens *B. burgdorferi, Paracoccidioides brasiliensis* and *Pneumocystis carinii* (38, 39, 41, 52).

AfEno1 is expressed on the surface of swollen conidia and hyphae, but not on resting conidia. In contrast, Aspf2, the second *A. fumigatus* factor H- and plasminogen ligand which is the homolog of the *C. albicans* immune evasion protein Pra1 is expressed on the surface of resting. The 74% stronger binding of plasminogen to swollen conidia, indicates that *A. fumigatus*, similar to *C. albicans* expresses additional plasminogen ligands (34).

Enolase is found at several sites and this moonlighting protein displays location specific functions. Cytoplasmic enolase catalyzes the conversion of 2-phosphoglycerate to phosphoenolpyruvate, surface exposed enolase binds human immune regulators and secreted enolase mediates immune evasion (42, 53). Enolase proteins from these pathogenic microbes lack typical signal-peptides, indicating that these proteins are secreted by an novel, unconventional mechanism (54). It is also unclear how enolase is anchored or integrated into the membrane or cell wall of *A. fumigatus* and of other pathogenic microbes. However, fungal moonlighting proteins including enolase are present in extracellular vesicles released by *C. albicans* (55). But, to the best of our knowledge, whether *A. fumigatus* releases any extracellular vesicles and the proteome of such extracellular vesicles has not yet been described.

Plasminogen together with complement components and regulators including factor H FHL-1 are present in the bronchoalveolar lavage fluids (7, 56). Thus, by reaching the alveoli and by hijacking these host proteins, *A. fumigatus* can control human complement attack and can use plasmin for cell damage and tissue destruction. Plasmin, the active protease, attached to swollen conidia inhibit complement, damage human epithelial cells, and degrade the ECM matrix. Such tissue destruction allows invasion and dissemination. Given these central features in immune evasion, enolase is considered *A. fumigatus* a central virulence factors.

At present two factor H ligands are identified from *A. fumigatus*, AfEno1 and Aspf2 which both are also identified as allergens (34). Sera from patients with ABPA have AfEno1 reacting IgE antibodies (53, 57, 58). AfEno1 modulates T cell response in healthy human individuals (53), but how exactly AfEno1 sensitizes the allergic T cells is unknown. For *C. albicans* four factor H binding proteins are so far identified i.e., pH-regulated antigen 1 (Pra1), glycerol-3-phosphate dehydrogenase 2 (Gpd2), high-affinity glucose transporter 1 (Hgt1), and phosphoglycerate mutase (Gpm1) (35). The Gram positive bacterium *S. aureus* has two identified factor H ligands, serine–aspartate repeat protein E (SdrE) and *Staphylococcus aureus* binder of IgG (Sbi) (59, 60). For *Streptococcus pneumoniae* two factor H ligands, i.e., Tuf from also recruits plasma regulators and PspC are identified (16). *Borrelia burgdorferi* expresses five surface expressed complement regulators acquiring surface proteins, CRASP-1–CRASP-5 and which confers *Borrelia* resistant to host complement attack (61, 62).

*C. albicans* expresses multiple plasminogen binding proteins, and over 12 plasminogen binding proteins are identified, i.e., pH-regulated antigen 1 (Pra1) (35), phosphoglycerate mutase (Gpm1), glycerol-3-phosphate dehydrogenase 2 (Gpd2), transcription elongation factor (Tef1), enolase (Eno1) (42), alcohol dehydrogenase (Adh1), glyceraldehyde-3-phosphate dehydrogenase (Tdh3), thiol-specific antioxidant-like protein 1 (Tsa1), phosphoglycerate kinase (Pgk1), fructose bisphosphate (Fba1), catalase (Cta1), and aldolase (35, 63–65). Expression of several plasma regulator-binding proteins by pathogens can be extrapolated to *A. fumigatus*.

In conclusion, AfEno1 from *A. fumigatus* is a moonlighting protein and allergen. AfEno1 surface expression is developmentally regulated. AfEno1 is expressed on the surface of swollen conidia and hyphae but not on resting conidia. Surface-exposed AfEno1 binds four human complement regulators which mediate/contribute to fungal survival in an immunocompetent host. AfEno1 hijacks the human zymogen plasminogen and tPA; activated plasmin damages human lung epithelial cells and degrades EMC proteins, thereby allowing tissue penetration, colonization, and dissemination. Given these multifaceted features, AfEno1 may represent a therapeutic target to fight *A. fumigatus* infection.

### Statistical Analysis

Statistical analyses of unpaired *t*-tests were performed with Prism 5 (GraphPad Inc., La Jolla, CA, USA). Differences with *P* < 0.05 were considered significant, and significance between two groups is shown as ^*^P < 0.05, ^**^P < 0.01, ^***^P < 0.001.

## Data Availability Statement

All datasets generated for this study are included in the article/Supplementary Material.

## Ethics Statement

The studies involving human participants were reviewed and approved by University Hospital Jena. The patients/participants provided their written informed consent to participate in this study.

## Author Contributions

PD, NB, PZ, CS, and AB designed and planned experiments. AS-R, MF, PZ, CS, and AB analyzed data. AS-R, PD, SD, NK, IS, and DW performed experiments. SD, PD, AB, PZ, CS, and MF evaluated data. PD, PZ, CS, MF, and NB wrote the manuscript.

### Conflict of Interest

The authors declare that the research was conducted in the absence of any commercial or financial relationships that could be construed as a potential conflict of interest. The handling editor declared a past co-authorship with one of the authors PZ.

## Supplementary Material

Supplementary Figure 1 Expression and purification of recombinant AfEno1 protein *A. fumigatus* AfEno1 was cloned, expressed in *E. coli*, and purified by Ni-NTA chromatography. **(A)** Proteins were separated by SDS-PAGE and visualized by Silver stain. S represents supernatant after IPTG induction (lane 1). FT is flow through after Ni-NTA column (lane 2), and W1 and W2 are washes (lane 4–5). E1 and E2 show elution fractions (lanes 6–8). The elution fractions were pooled and different amounts of protein was separated by SDS-PAGE, transferred to a membrane. Recombinant AfEno1 was identified by Western blotting with either **(B)** monoclonal anti-his antibody (lanes 1–2) or **(C)** AfEno1 antiserum (lanes 1–2).

Supplementary Figure 2 Recombinant AfEno1 binds to plasma regulators. **(A)** AfEno1 binds to Factor H dose-dependently. Factor H was immobilized onto a microtiter plate, and AfEno1 at increasing amounts was added. After washing, bound AfEno1 was detected with rabbit AfEno1 antiserum. AfEno1 did not bind to gelatin. **(B)** AfEno1 binds to FHL-1 dose-dependently. AfEno1 at indicated amounts was added to immobilized FHL-1, and attached AfEno1 was detected with rabbit AfEno1 antiserum. FHL-1 showed no binding to gelatin. **(C)** Serum-derived Factor H binds to AfEno1 dose-dependently. NHS at increasing amounts was added to immobilized AfEno1. After washing, bound Factor H was detected with goat human Factor H antiserum. **(D)** C4BP binding to AfEno1 was assayed by ELISA. AfEno1 was immobilized onto a microtiter plate overnight, and C4BP at increasing amounts was added. After washing, bound C4BP was detected with goat human C4BP antiserum. C4BP showed no binding to gelatin. **(A–D)** show mean values ± SD from three separate experiments.

## Abbreviations

FHL-1: factor H-like protein 1
ELISA: enzyme-linked immunosorbent assay
DPBS: Dulbecco's phosphate-buffered saline
RT: room temperature
SCRs: short consensus repeats
tPA: tissue-type plasminogen activator
ε-ACA: ε-aminocaproic acid.
