# Supplementary figures and images for: Enolase From Aspergillus fumigatus Is a Moonlighting Protein That Binds the Human Plasma Complement Proteins Factor H, FHL-1, C4BP, and Plasminogen
Source: Front Immunol. 2019 Nov 22;10:2573. doi: 10.3389/fimmu.2019.02573 (PMC6883375; doi:10.3389/fimmu.2019.02573)

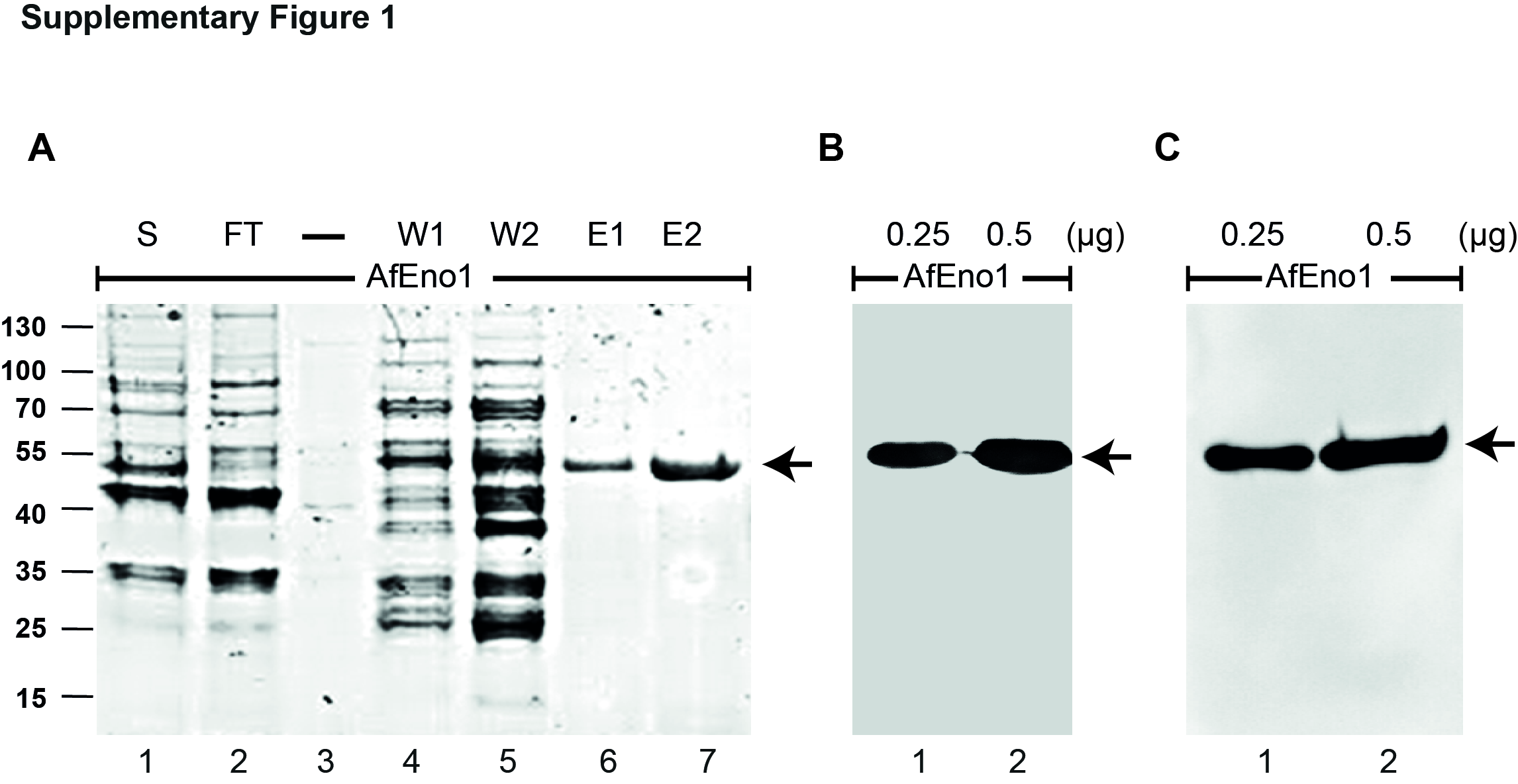

Supplement: Supplementary Figure 1 — Expression and purification of recombinant AfEno1 protein A. fumigatus AfEno1 was cloned, expressed in E. coli, and purified by Ni-NTA chromatography. (A) Proteins were separated by SDS-PAGE and visualized by Silver stain. S represents supernatant after IPTG induction (lane 1). FT is flow through after Ni-NTA column (lane 2), and W1 and W2 are washes (lane 4–5). E1 and E2 show elution fractions (lanes 6–8). The elution fractions were pooled and different amounts of protein was separated by SDS-PAGE, transferred to a membrane. Recombinant AfEno1 was identified by Western blotting with either (B) monoclonal anti-his antibody (lanes 1–2) or (C) AfEno1 antiserum (lanes 1–2). [file Image_1.TIF]

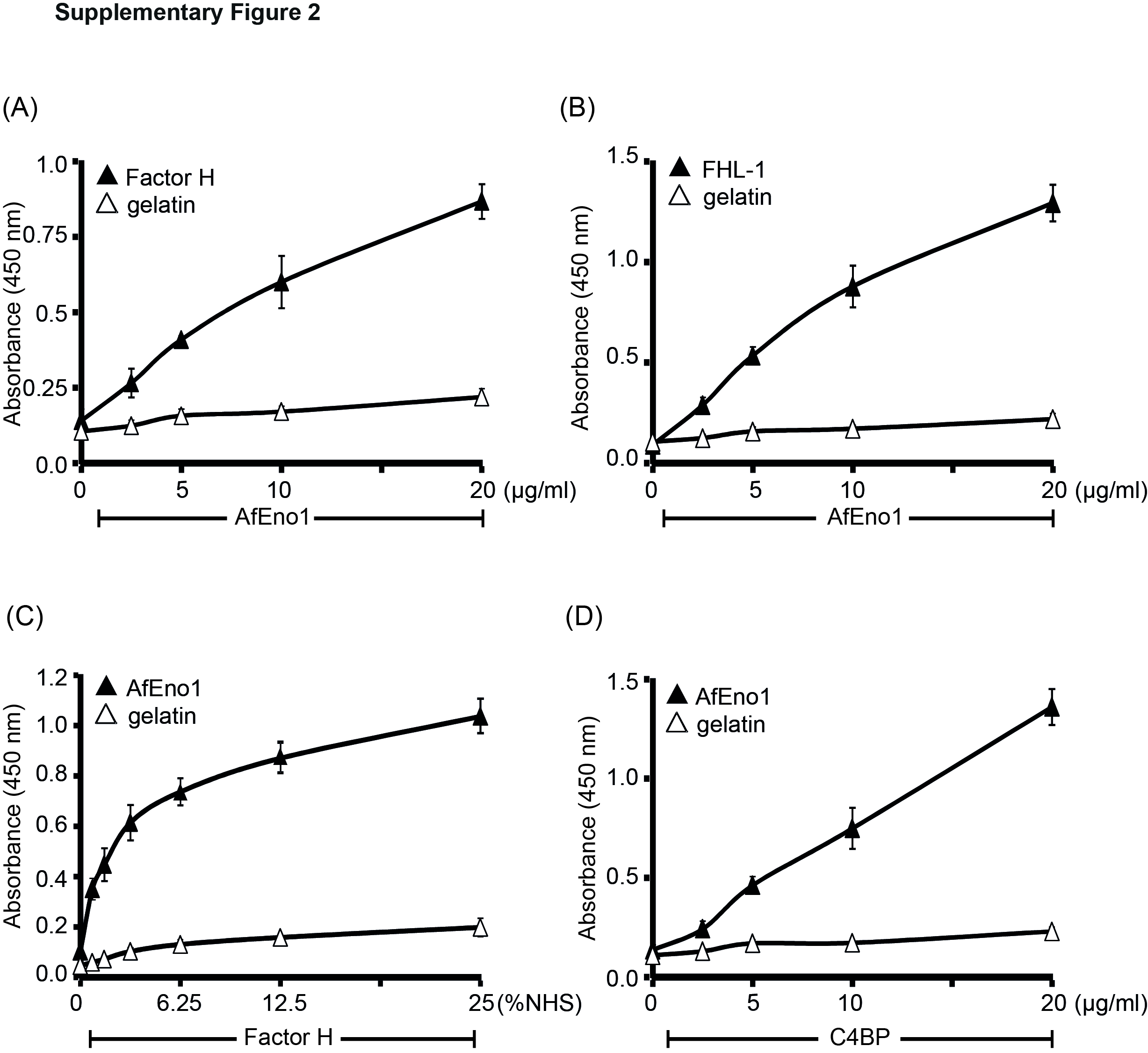

Supplement: Supplementary Figure 2 — Recombinant AfEno1 binds to plasma regulators. (A) AfEno1 binds to Factor H dose-dependently. Factor H was immobilized onto a microtiter plate, and AfEno1 at increasing amounts was added. After washing, bound AfEno1 was detected with rabbit AfEno1 antiserum. AfEno1 did not bind to gelatin. (B) AfEno1 binds to FHL-1 dose-dependently. AfEno1 at indicated amounts was added to immobilized FHL-1, and attached AfEno1 was detected with rabbit AfEno1 antiserum. FHL-1 showed no binding to gelatin. (C) Serum-derived Factor H binds to AfEno1 dose-dependently. NHS at increasing amounts was added to immobilized AfEno1. After washing, bound Factor H was detected with goat human Factor H antiserum. (D) C4BP binding to AfEno1 was assayed by ELISA. AfEno1 was immobilized onto a microtiter plate overnight, and C4BP at increasing amounts was added. After washing, bound C4BP was detected with goat human C4BP antiserum. C4BP showed no binding to gelatin. (A–D) show mean values ± SD from three separate experiments. [file Image_2.TIF]
